# Attenuation of Self-Generated Tactile Sensations Is Predictive, not Postdictive

**DOI:** 10.1371/journal.pbio.0040028

**Published:** 2006-01-17

**Authors:** Paul M Bays, J. Randall Flanagan, Daniel M Wolpert

**Affiliations:** **1**Sobell Department of Motor Neuroscience, Institute of Neurology, University College London, United Kingdom; **2**Department of Psychology and Centre for Neuroscience Studies, Queen's University, Kingston, Ontario, Canada; **3**Department of Engineering, University of Cambridge, Cambridge, United Kingdom; Brandeis UniversityUnited States of America

## Abstract

When one finger touches the other, the resulting tactile sensation is perceived as weaker than the same stimulus externally imposed. This attenuation of sensation could result from a predictive process that subtracts the expected sensory consequences of the action, or from a postdictive process that alters the perception of sensations that are judged after the event to be self-generated. In this study we observe attenuation even when the fingers unexpectedly fail to make contact, supporting a predictive process. This predictive attenuation of self-generated sensation may have evolved to enhance the perception of sensations with an external cause.

## Abstract 2

Sensory perception is influenced by whether sensations are self- or externally generated. This study suggests a process in which predicted sensory consequences of an action are responsible for the difference in perception.

## Introduction

Differential processing of self- and externally generated sensory signals appears to be a fundamental means by which organisms interpret their environment, and has been observed across a variety of species and sensory systems [1–4]. In humans, self-generated tactile sensations are perceived as weaker than the same stimuli externally imposed [5–7]. This sensory attenuation has generally been thought to result from a predictive mechanism. According to this hypothesis, when a motor command is generated, an “efference copy” of this command is used to predict the sensory consequence of the action [8–10]. This predictable component is removed from the incoming sensory signals, thereby increasing the relative salience of sensations with an external cause. In such a mechanism, the sensory signal is altered online and the original sensory input is no longer available for further processing.

Although the human psychophysical data are consistent with a predictive mechanism, they are equally consistent with a reconstructive, or postdictive, mechanism. A postdictive mechanism is one in which the percept of a sensory event is constructed from sensory information received around the time of the event [11–13]. In this mechanism, the original sensory input is available for a period after the event and its processing can depend substantially on other events that occur in close temporal proximity. The “cutaneous rabbit” illusion [14] provides an example of postdiction in the tactile modality. A rapid series of taps applied to the hand are reported to “hop” up the arm if immediately followed by one or more taps near the elbow, but remain localized at the hand if no subsequent taps are delivered. Thus, the processing of the initial taps depends on the subsequent taps. If a postdictive mechanism is responsible for tactile sensory attenuation, a given sensation will be identified as either self- or externally generated on the basis of all the sensory events that occurred at around the same time. For example, a tap on a passive finger may be more likely to be judged to be self-generated, and therefore attenuated, if the active or tapping finger receives tactile feedback at the same time. In this study we distinguish between predictive and postdictive hypotheses by constructing a stimulus that a postdictive mechanism will identify as externally generated but that is nonetheless predictable on the basis of the motor command, and hence will be attenuated by a predictive mechanism.

## Results

Participants in one group (A) were required to judge which of two taps delivered sequentially to their left index finger was harder. The first tap (test tap) was of fixed magnitude (2.7 N), whereas the size of the second tap (comparison tap) was varied from trial to trial. A maximum-likelihood procedure was used to determine the perceived magnitude of the test tap: that is, the ratio of the comparison tap to the test tap at which the two taps were perceived as equal (see Materials and Methods). On contact trials, which made up the majority of trials, the test tap was triggered with minimal delay by the participants tapping with their right index finger on a force sensor fixed above their left index finger (Figure 1A, top). This situation simulates directly tapping onto one's own finger through a solid object. We have previously shown that sensation of the test tap is substantially attenuated in this condition [7]. Delay trials were identical except that the test tap was delayed by 500 ms relative to the participant's tap on the force sensor. In our previous study, we showed that attenuation in the passive finger is closely synchronized with the contact time of the active finger and that attenuation was not observed when the test tap is delayed by 300 ms. The delay trials therefore provided a baseline measure, without attenuation, of the participants' performance on the comparison task, taking into account any small response bias or “time error” [15] that might influence the comparison. As expected, the perceived magnitude of the test tap on contact trials (mean 0.71, standard error [SE] 0.08; Figure 1B) was significantly reduced compared to the baseline set by the delay trials (mean 0.95, SE 0.04; F_1,9_ = 6.4; *p* = 0.032).

**Figure 1:**
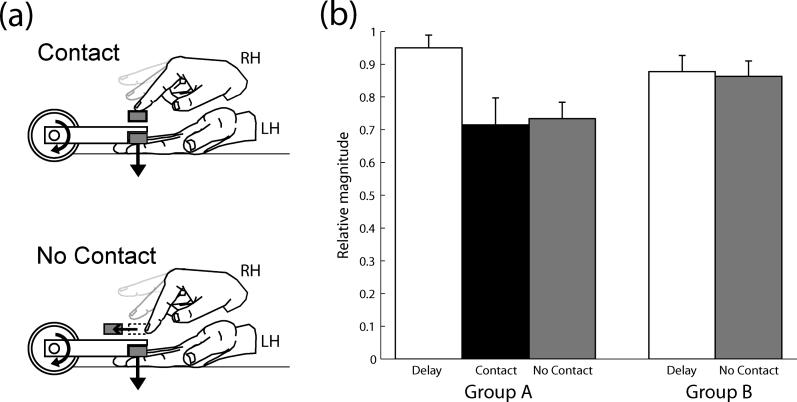
Experimental Task and Results (A) Schematic of the apparatus and task. On contact trials (top), in response to an auditory go signal, participants produced a brief force pulse with their right index finger on a force sensor fixed above their left index finger. A similar force pulse was delivered to the left index finger by a torque motor. On no-contact trials (bottom), the force sensor was moved at the start of the trial so that participants made a tapping movement with their right index finger but did not make contact. (B) Mean relative magnitude of the comparison tap to the test tap at the point of perceptual equality as a function of trial type and participant group. Error bars represent ±1 SE.

On infrequent no-contact trials, unknown to the participant, the force sensor was moved at the start of the trial, so that at the go signal the participant made a tapping movement, but made no contact (Figure 1A, bottom). On these trials, the test tap was triggered when the participant's finger passed through the position at which contact would have been made with the force sensor had it not been moved. The perceived magnitude of the test tap on these trials was substantially reduced (mean 0.73, SE 0.05; Figure 1B) compared to delay trials (F_1,9_ = 15.5; *p* = 0.0034), and did not differ significantly from that seen in contact trials (F_1,9_ = 0.11; *p* = 0.75). This is consistent with the action of a predictive attenuation mechanism: As the test tap in these trials occurred at the same time as it would have in a contact trial, a predictive mechanism would correctly predict the tap and attenuate it. In contrast, if attenuation were the result of a postdictive mechanism that integrates sensory input from around the time of the tap, then on these trials the test tap would not be recognized as self-generated (because no contact occurred) and attenuation would not be observed. We can therefore conclude that a postdictive mechanism is not responsible for tactile sensory attenuation.

This interpretation depends on the assumption that the sensation of contact in the active finger is necessary for attenuation in the passive finger. It may be that the attenuation mechanism comes to associate the occurrence of the test tap with a particular movement or location of the active finger. To test this possibility, a second group of participants (B) took part in an experiment that was similar but in which the active finger never made contact with the force sensor. The test tap was triggered as the finger passed through the position at which contact would have been made in the first experiment either immediately (no-contact trials, which made up the majority of trials), or after a 500-ms delay (delay trials). In contrast to the first group, the perceived magnitude of the test tap did not differ significantly between these two types of trial (F_1,9_ = 1.1; *p* = 0.31; Figure 1B). A significant group-condition interaction (F_1,18_ = 12.7; *p* = 0.0022) confirmed the difference between the two groups. We can conclude that a consistent relationship between motor activity in one finger and sensation in another finger is not sufficient to induce attenuation.

An examination of individual participant data shows that the responses of the majority of participants were in accordance with the group mean results described above. Of the ten participants in group A, seven showed a reduction of at least 10% in perceived magnitude on contact trials compared to delay trials. Of these participants who showed clear attenuation, all but one also showed a reduction of at least 10% on no-contact trials. In contrast, in group B the difference in perceived magnitude between no-contact and delay trials was less than 10% in every participant.

## Discussion

We have found strong evidence to suggest that attenuation of self-generated tactile sensation results from a predictive mechanism. When one finger made a tapping movement above a finger of the other hand, sensation in the passive finger was attenuated only when contact was expected between the fingers. The level of attenuation observed when contact was expected was the same whether or not the contact actually occurred. These results are inconsistent with a postdictive mechanism, which would have access to the actual sensory feedback in determining the level of attenuation. Our results are instead consistent with the action of a predictive mechanism—an online process that predicts self-generated sensory events on the basis of planned motor activity and attenuates the incoming sensory stream so as to reduce the sensory salience of those events.

The absence of attenuation in participants in group B, who experienced taps in the passive finger but never experienced a contact event in the active finger, is an important result for several reasons. First, it serves as a control for effects of divided attention. Participants in group A had to attend to two different tasks at the same time: generating a tap with the active finger and judging a sensation in the passive finger. This division of attention may be less pronounced in delay trials, where the two tasks were separated by 500 ms, than in contact and no-contact trials, where the two tasks were simultaneous. This difference in attentional demand could conceivably be responsible for the difference in magnitude judgment between these trial types. However, group B was essentially identical to group A with respect to attention: participants had to generate tapping movements with amplitudes and velocities matched to those made by participants in group A, and the judgment task in the passive finger was identical in both groups. If divided attention were responsible for the differences in magnitude judgment between trials with and without a delay in group A, the same differences would be seen between no-contact and delay trials in group B. Because no such differences were observed, we can conclude that the reduction in perceived magnitude seen in group A was due to a specific attenuation mechanism and not the attentional demands of the task.

Second, group B served to rule out a postdictive mechanism based only on motion cues. In group A, attenuation was observed in the passive finger even when the active tap failed to make contact. Although this result was precisely what is expected from a predictive mechanism, it is also consistent with a postdictive mechanism that relies only on motion cues and ignores tactile cues. Such a hypothetical mechanism would attenuate the sensation of the tap in the passive finger simply due to its temporal and spatial proximity to the movement of the active finger. However, such a mechanism would also produce attenuation in the no-contact trials in group B, and this was not observed. We can conclude that postdictive processes do not play a significant role in the attenuation of self-generated tactile sensation.

The attenuation that we have observed in this study is distinct from the increase in detection threshold to cutaneous stimuli observed in a moving effector [16] in that it is present in a passive digit and is synchronized to the expected time of self-contact [7]. Moreover, a movement-related increase in threshold is also seen in advance of a passive movement that occurs at an unpredictable time [17], suggesting it involves a postdictive or masking mechanism rather than the kind of predictive mechanism demonstrated in this study.

The neural mechanisms of predictive cancellation have been extensively studied in the electrosensory system of the electric fish [1]. Electric fish possess both electroreceptors for sensing current and electric organs driven by motor commands for discharging current. To prevent the discharge of a fish's own electric organs interfering with its ability to sense its surroundings, the self-generated component is removed from the output of electrosensory cells. There are two key features of this cancellation mechanism. First, a prediction of the expected sensory input is generated based on an efference copy of the motor command; this prediction is subtracted from the activity of neurons that receive electrosensory input [18]. This predictive cancellation is adaptive: in fish whose electric organs have been blocked by curare, a consistent pairing of an electrical stimulus with the motor command leads to cancellation of the novel input. Second, cancellation is observed for sensory inputs that are predictable only on the basis of incoming sensory information such as proprioception. For example, pairing an electrical stimulus with a particular phase of a passive sinusoidal tail bend is sufficient to produce sensory cancellation [19,20]. These studies show that, provided the sensory input is predictable based either on the descending motor command or on proprioceptive feedback, cancellation occurs. In contrast, our study shows that in the human somatosensory system a highly specific set of events has to occur for predictive cancellation to take place. A consistent pairing of a tactile stimulus with a particular angle of active finger flexion was not sufficient to produce attenuation, despite the tactile input's predictability based both on the motor command and on proprioceptive feedback. This suggests that the cancellation mechanism in the human somatosensory system is not identical to that in electric fish. Instead, attenuation of self-generated tactile sensation results from a mechanism that depends on prediction of a specific event—contact—rather than arbitrary predictable stimuli or postdictive reconstruction.

Predictive cancellation in electric fish occurs in regions of the hindbrain that are analogous to the human cerebellum. Several computational models of the human sensorimotor system have also proposed a role for the cerebellum in predicting the sensory consequences of motor activity [21–23]. Such a role is supported by an fMRI study that examined the neural responses to tactile stimulation of the hand [24]. An externally generated tactile stimulus in the left hand produced activation in the contralateral primary and bilateral secondary somatosensory cortex. When participants instead actively generated the tactile stimulation with their right hand, the activation in the secondary somatosensory cortex was significantly reduced. A similar suppression of neural activity could underlie the attenuation observed in the current study when a finger of the right hand tapped on the left. Activity in the right anterior cerebellar cortex was also reduced when the stimulus was self-generated. This may reflect a role for cerebellar areas ipsilateral to the active hand in generating the predictive cancellation signal that is used to attenuate activity in sensory areas.

The ability of the nervous system to predict the sensory consequences of motor activity may have further uses in addition to its role in sensory attenuation (see [25] for a review). This kind of prediction may underlie our ability to mentally rehearse movements before carrying them out. Such prediction is also integral to proposed mechanisms that compensate for inaccuracies and delays in sensory feedback [22,26], and adjust motor output to suit the context of a movement [27].

## Materials and Methods

After providing written informed consent, 20 right-handed participants aged 18–40 took part in the experiment, ten in group A (four men, six women) and ten in group B (nine men, one woman). A local ethics committee approved the experimental protocols. Each participant rested his or her left index finger in a molded support beneath a force sensor mounted on a lever that was attached to a torque motor (Figure 1A). The right index finger was held above the left index finger in a support that constrained it to a single flexion-extension movement, and the angle of flexion was recorded by a rotary encoder. At an auditory go signal participants tapped with their right index finger (active finger) on a force sensor fixed above, but not in contact with, their left index finger (passive finger). Two taps (test tap followed by comparison tap) were delivered to the left index finger by the torque motor, separated by an interval of 700–1,200 ms. Participants indicated which of the two taps on the left index finger they perceived as harder by pressing one of two response buttons. Both taps had a fixed duration of 80 ms. The peak force amplitude of the comparison tap was varied across trials according to a maximum-likelihood procedure (see below) so as to find the amplitude at which it was perceived as identical to the test tap (which had a fixed amplitude of 2.7 N).

Each participant completed 50 sets each of six trials. For Group A, the majority of trials (four trials in each set of six) were contact trials. On these trials, the test tap on the left finger was triggered by the participant's active tap on the top force sensor with almost zero delay (CPU processing time and the dynamics of the torque motor inevitably introduced a small delay of approximately 11 ms); thus, for the participant the situation closely mimicked tapping directly on the left index finger with the right. Participants had been trained (during an earlier practice session) to produce a tap with an amplitude between 1.75 N and 3.5 N; during the experimental session, trials in which the amplitude of the participant's active tap fell outside of this range were rejected. On delay trials (one trial in each set), the test tap was delayed by 500 ms relative to the active tap. On no-contact trials (one trial in each set), the top force sensor was moved without the participant's knowledge (both force sensors were always hidden from view) prior to the go signal. This meant that when the go signal was given, the participant made a tapping movement but did not make contact with the top force sensor. On these trials the test tap was triggered when the right index finger passed through the flexion angle at which contact would have been made with the top force sensor. Group B underwent an identical protocol to group A, except that no contact was made with the force sensor on any trial. Thus, the majority (five trials in each set) were no-contact trials as described above, and on delay trials (one trial in each set) the test tap was triggered in the same way as in the no-contact trials, but with a 500-ms delay. Participants in this group were trained (in an earlier practice session) to make movements with similar amplitude and velocity to the movements made in no-contact trials by participants in group A. For this reason, trials with movement amplitude outside the 45–70 ° range or peak downward velocity outside the 550–1,000 °s^−1^ range were rejected. The motor output a participant had to generate to successfully complete a trial was therefore very similar in groups A and B. In both groups, the order of trials within each set was pseudorandomized.

At the end of each trial, the comparison tap amplitude and the participant's response on that trial were pooled with the data from all previous trials under the same condition. The data from each condition were fitted with a logistic function according to a maximum-likelihood procedure, and the response threshold (the 50% point on the fitted psychometric curve) was calculated to estimate the comparison tap amplitude that would make the test and comparison taps perceptually equal. For the first ten trials in each condition, a force amplitude was chosen from a uniform random distribution in the 0.5–5.5 N range, and this amplitude was used for the comparison tap on the next trial in the same condition. For subsequent trials, the comparison tap amplitude was chosen from a narrower range defined by the 1% and 99% points on the fitted psychometric curve. This procedure focused sampling in the region of the current estimate of the response threshold without overly restricting the range of amplitudes tested (the mean width of the final sampling range was 3.3 N). In subsequent analysis, the response threshold was calculated over all responses for each participant and trial condition. Within-participant and between-participant comparisons between trial conditions were made using repeated-measures analysis of variance.

## Abbreviation

SE: standard error
